# The Costs of Store Sales for Retail Workers

**DOI:** 10.3389/fpsyg.2020.536970

**Published:** 2020-11-06

**Authors:** Paul van der Laken, Susanne Beijer, Sanne Nijs, Marc van Veldhoven, Jaap Paauwe

**Affiliations:** ^1^ Vereniging Achmea, Zeist, Netherlands; ^2^ Department of Management and Organization, School of Business and Economics, Vrije Universiteit Amsterdam, Amsterdam, Netherlands; ^3^ Department of Human Resource Studies, Tilburg School of Social and Behavioral Sciences, Tilburg University, Tilburg, Netherlands

**Keywords:** work engagement, job characteristics, multilevel analysis, retail, store sales

## Abstract

In the context of economic stagnation and recession, retailers face fierce competition and experience enormous pressure to increase their sales. In this study, we focus on the potential costs of higher store sales for retail workers by examining its effect on work engagement. Drawing on work intensification literature and the job demand-resources model, we study how job variety and workload, two job characteristics, mediate the relationship between store sales and engagement. Store revenue data and survey data of 525 sales employees, embedded in 110 stores of a large Dutch retail organization were used, to perform mixed models analyses. The analyses demonstrate that store sales is negatively related to job variety and positively related to workload. In turn, job variety positively affects work engagement, while workload negatively affects work engagement. Based on multi-source, multilevel data it is thus shown that there are negative effects of store sales in retail. More insight is created into the job characteristics that explain the negative link between store sales and engagement. As it is empirically demonstrated that there are indeed costs associated with improved performance in retail, it is crucial that organizations ensure investments in maintaining resourceful work environments.

## Introduction

The economic stagnation and recession of European retail markets of the last decade (CBS, 2014) has intensified competition between retailers. As a consequence retail businesses feel pressured to increase store sales in order to remain profitable. Retailers seem to be convinced that increasing sales is the way forward. However, studies suggest that management interventions aimed at increasing sales have far-reaching consequences for employee’s well-being and attitudes (Godard, 2001, 2004; Van de Voorde et al., 2012).

Understanding the relationship between store sales and employee outcomes is crucial for ensuring a sustainable future and a number of studies have started to examine this issue by, for example, looking at how sales affects customer-oriented attitudes (Yoo and Arnold, 2016). A topic that has gained limited attention in this regard so far is work engagement, which has been defined as a positive, fulfilling emotional state of work-related well-being (Schaufeli et al., 2002). It is frequently measured and studied by work and organizational psychologists as it has shown to relate to lower turnover, higher customer loyalty (Harter et al., 2002), and higher in- and extra-role performance (Christian et al., 2011). Two competing theories circulate in the literature regarding the effect that store sales have on employees’ engagement. On the one hand, scholars propose that well-performing organizations share their success with their employees (Schneider et al., 2003), who in turn reciprocate by demonstrating high levels of engagement (Gouldner, 1960; Adams, 1965). On the other hand, work intensification theory (e.g., Godard, 2001, 2004), suggests that high sales can also have negative consequences for work engagement via work intensification.

It currently remains unclear how pressures for increasing sales affect workers’ well-being. Given the competing theories, it is important to consider contextual factors that might influence the effect store sales have on engagement. One factor that might play a role in understanding the relationship between store sales and engagement is the nature of work. In some businesses, increased sales may lead to investments in the workforce, creating more resourceful and motivating jobs. In other businesses, however, increased sales may have negative effects, as it requires employees to demonstrate specific behaviors or exert additional effort.

The current study aims to gain insight in the effects of store sales in the context of a Dutch retail chain by examining whether higher levels of store sales are indirectly associated with certain costs in terms of decreased employee engagement (van der Laken, 2014). This study aims to provide insight into the processes through which store sales affects work engagement in the retail context, since, we currently have limited understanding of the mechanisms behind the sales-engagement relationship. More specifically, the current study contributes to the literature as based on work intensification and job demands-resources literature by examining two distinct processes. More specifically, it is studied how sales affects work engagement via workload and job variety. With this study, we thus further unravel the link between organizational outcomes and employee outcomes (Paauwe and Farndale, 2017) by looking at how two work characteristics mediate this relationship. By taking such a multilevel approach, we unravel unexplored tensions between interests of employees and organizations in the retail sector and show how store sales can potentially harm work engagement levels. Also, by making use of both subjective employee data and objective store performance data the effects of an objective indicator on work engagement are validated.

## Theoretical Background

### Job Variety, Workload, and Work Engagement

Work engagement has surged as a hot topic among managers as it is identified as a driver of increased organizational performance (Harter et al., 2002; Rich et al., 2010; Christian et al., 2011). The academic literature has also extensively studied work engagement, and the job demands-resources model (JDR; Demerouti et al., 2001; Van den Broeck et al., 2010; Bakker and Demerouti, 2017) has explored two ways in which job characteristics influence work engagement. While job resources on the one hand have motivational potential, job demands, on the other hand, lead to health issues and strain, which relate negatively to motivation (Bakker and Demerouti, 2006, 2017). Accordingly, it can be expected that job resources have a positive influence on engagement, while job demands have a negative influence on work engagement (Lesener et al., 2019).

In the context of retail, Yoo and Arnold (2016) have shown that job demands can weaken the positive effects of job resources on customer-oriented attitudes. Salanova et al. (2005) applied the JDR model to the service industry and showed that organizational resources affect service climate, which is in turn related to employee performance and customer outcomes. On a more general level, a wide range of studies have provided empirical support for the causal relation between job resources and work engagement (e.g., Schaufeli et al., 2009). Especially task-related job resources, such as job variety, have large motivational potential (Halbesleben, 2010). Firstly, job variety fosters employee growth and development, which enhances the intrinsic motivation of employees, the latter being a strong predictor of work engagement (Leiter and Maslach, 2010). Secondly, job variety provides employees with extrinsic motivation by aiding them in achieving their work goals (Ryan and Deci, 2000; Schaufeli and Bakker, 2004; Schaufeli et al., 2009). Thirdly, job variety fulfills basic human needs, providing employees with a sense of competence, relatedness, and autonomy (Ryan and Deci, 2000).

Simultaneously, poorly designed jobs and continuous, excessive job demands can exhaust and strain employees leading to health issues (Bakker and Demerouti, 2006). The high effort employees need to show in order to deal with job demands can result in feelings of decreased energy and vigor (Van den Broeck et al., 2010; Mauno et al., 2019). Crawford et al. (2010) suggest that individual and contextual characteristics play a large role in how these job demands are appraised. Applying this to the current retail context, it is expected that in this industry employees will most likely experience workload as a job hindrance. The retails sector is characterized by limited discretion and a transactional nature of employment relations. This context makes that the increased workload will most likely be perceived as hindering instead of challenging by employees, which exerts a negative effect on work engagement.

*Hypothesis* 1: Job variety is directly positively associated with work engagement.

*Hypothesis* 2: Workload is directly negatively associated with work engagement.

### Store Sales and Job Characteristics

Work intensification theory argues that organizations aiming for high sales revenue are likely to increase the workload of their employees in order to achieve organizational goals (Godard, 2001, 2004; Allan and Lovell, 2003) but at the same time, these organizations also make work less varied in certain specific contexts. Previous studies already demonstrated that this negatively affects health-related well-being (Van de Voorde et al., 2012). In this study context (i.e., retailers), higher levels of sales are mainly caused by customer growth in demand which means that more frequent restocking and/or more voluminous check-outs at the register are required. This means that employees need to meet higher demands when sales go up, mainly in the form of value-adding tasks. Most likely, these employees experience less job variety because tasks, like familiarizing new colleagues, extensive customer service, reorganizing store layout, and other non-essential tasks are surpassed for direct revenue-generating tasks such as working the register and restocking. It is thus argued that on a workday where high sales figures are reached, more customers come into the store and employees therefore need to work at the register a larger proportion of their time, and fill the shelves more frequently than when sales are low. Consequently, employees are forced to focus on only two tasks of their job description, instead of being able to carry out a variety of duties. This means that there is an immediate link between sales and job variety in this specific context. Based on work intensification theory, we hypothesize that store sales negatively affect employees’ job resources in a retail context. More specifically, higher sales result in intensification of work, only leaving room for carrying out a small array of tasks and thus lowering job variety.

*Hypothesis* 3: Store sales is directly negatively associated with job variety.

In addition to this negative effect on job variety, previous studies have also shown that store sales positively relates to workload (Janssen, 2001; Van de Voorde et al., 2016). In retail, sales is primarily a function of the amount and value of products sold and, as such, an increase in the amount and value of products sold will lead organizations to demand more effort of their employees (Barker, 1993; Janssen, 2001). More frequent and/or more voluminous checkouts at the register will namely demand more effort from employees, and will result in the need to restock the shelves. The more frequent checkouts and restocking activities thus result in more work activities, increasing the workload of store employees. This is in line with work intensification theory, which argues that aiming for better financial performance will intensify work and put additional pressure on employees (Godard, 2001, 2004; Green, 2002; Allan and Lovell, 2003). Empirical work indicates that high sales contexts bring along additional job demands and signal that additional effort is expected from employees (Butts et al., 2009; Wood and De Menezes, 2011). Moreover, employees are more likely to perceive little resources to cope with these heightened work demands. The economic climate (CBS, 2014) strengthens the intensification process as higher levels of sales are not likely to be accompanied with staff increases or other investments aimed at coping with these demands. Based on the above, we expect store sales to positively relate to workload in the context of retail.

*Hypothesis* 4: Store sales is directly positively associated with workload.

### The Mediating Role of Job Variety and Workload

The above presents two indirect pathways from store sales to work engagement. Both job variety and workload are expected to mediate the negative effect of store sales on work engagement of retail employees.

In the current study, we base ourselves on work intensification theory, which suggests that high sales can result into more stressful work environments (e.g., Allan and Lovell, 2003) and that these contexts can become too demanding for employees (Barker, 1993; Janssen, 2001). More specifically, work intensification theory argues that organizations aiming for high sales are likely to increase the workload of their employees in order to achieve their organizational goals (Godard, 2001, 2004; Allan and Lovell, 2003). Although, to date, these negative processes have only been demonstrated to affect health-related well-being (van de Voorde et al., 2012), they might ultimately cross over to work engagement.

Work intensification theory fits the current study, which focuses on the retail context. In the context of fierce competition in retail and the prevalence of strategies of cost containment and workforce reduction, it can be expected that high store sales levels will decrease work engagement due to the additional workload employees experience in maintaining the high sales standards (Barker, 1993). In this context, higher store sales requires more voluminous and/or more frequent check-outs at the register and immediately impacts the amount and type of tasks that need to be performed. Having to process a larger number of transactions and having to frequently restock shelves requires increased efforts of sales employees. To achieve high store sales, retail stores need to increase these sales without concurrent investments in human capital. This urges current employees to focus on the value-adding elements of their job. As a consequence, sales employees are expected to experience less variety in their work tasks, resulting in lower intrinsic motivation and engagement (Demerouti et al., 2001). Second, high store sales entails a more demanding environment for sales employees. A larger number of customers need to be served, more products need to be sold, and shelves to be stocked more frequently. This results in straining and demotivating work (Demerouti et al., 2001; Van den Broeck et al., 2010). Accordingly, it is expected that in this specific context increased sales will negatively affect work engagement via less job variety and more workload.

*Hypothesis* 5: Store sales have an indirect negative effect on work engagement via less job variety.

*Hypothesis* 6: Store sales have an indirect negative effect on work engagement via more workload.

**Figure 1:**
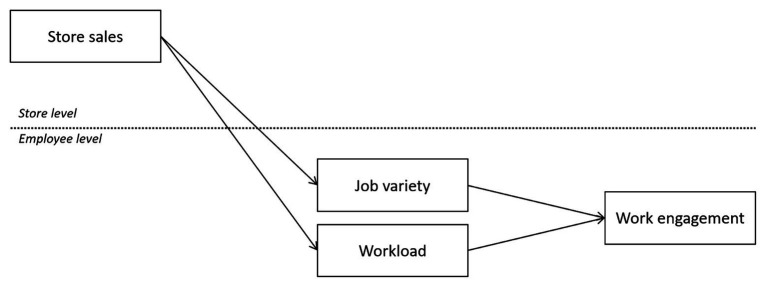
Conceptual model.

The hypothesized relationships are visualized in Figure 1.

## Materials and Methods

### Procedure and Sample

Multilevel data were collected in branches of a retail organization across the Netherlands. In October 2013, sales employees in almost 300 stores were invited to participate in the study. Participation was voluntary, anonymous, and during work hours. Around 731 out of 1,942 sales employees (37.64%) participated. Because up to 22 sales employees worked in a single store (*μ* = 6.23; *SD* = 2.753) and store generalizability had to be guaranteed to some extent, only data of stores with more than three complete responses of sales employees were used in the present analysis. These data were coupled to the objective store sales data provided by the organization. Once store sales and employee data were merged, the final dataset consisted of 525 sales employees embedded in 110 stores, resulting in a total response rate of 27.03%.

Of these 525 respondents, 98.7% were female. This was an accurate representation of the population, since 98.5% of the total working population in this retail organization was female. Most of the sales employees in the sample had a lower vocational educational background (43.4%). The largest proportion of employees belonged to the age group between 20 and 30 years old (39.6%), while 31.1% of the respondents were younger than 20 years and 29.3% respondents were older than 30 years. With respect to tenure; 17.9% had worked at the organization for less than a year, while 22.1% of the respondents had been employed for over 10 years. Finally, the majority of the sample had a permanent contract (57.0%) and worked less than 21 h per week (58.3%).

Ethical review and approval was not required for the study on human participants in accordance with the local legislation and institutional requirements. Written informed consent for participation was not required for this study in accordance with the national legislation and the institutional requirements. No potentially identifiable human images or data is presented in this study. No animal studies are presented in this manuscript. The datasets generated for this study will not be made publicly available as the organization studied does not allow the data to be publicly available.

### Measures

#### Work Engagement

The level of work engagement of sales employees was assessed using a shortened version of the Utrecht Work Engagement Scale, which consists of nine items (Schaufeli and Bakker, 2003). Respondents were asked to indicate on a seven-point scale ranging from one (never) to seven (always) how frequently statements applied to them. An example item is “At work, I feel healthy and strong.” Cronbach’s Alpha of the scale is 0.951.

#### Store Sales

Store sales reflect the revenue of a store, with higher sales reflecting more customers in the stores buying more products. Objective, financial store sales figures were provided by the organization. The sales per employee worked hour was calculated by taking the total revenue of a store during the last quarter of 2013 and dividing it by the total number of hours worked by that store’s sales employees during that last quarter. Explicitly the figures of the last quarter were selected because they constituted the financial figures during the timeframe in which the survey data were collected. This performance indicator was considered most suitable because it takes variation in store size into account (c.f. Piening et al., 2013), and as such was comparable across the unequally sized branches. Due to confidentiality reasons, exact financial data cannot be presented here.

#### Job Variety

Job variety is assessed with four items (van Veldhoven et al., 2002). Respondents were asked to indicate how frequently statements applied to them on a four-point scale ranging from one (never) to four (always). An example of an item was “Do you have enough variety in your work?” Cronbach’s Alpha was 0.786.

#### Workload

Workload was operationalized using six items of a shortened version of the VBBA scale of pace and amount of work (van Veldhoven and Meijman, 1994; van Veldhoven et al., 2002). It assesses the extent to which respondents feel pressured in the performance of their work activities. Respondents were asked to indicate on a four-point scale ranging from one (never) to four (always) how frequently statements applied to them. An example item is “Do you have to hurry?” Cronbach’s Alpha was 0.855.

For every scale, the sum of the item scores was calculated and then standardized to obtain standardized coefficients directly from the analyses (Hofmann and Gavin, 1998; Enders and Tofighi, 2007). In this way, the effects of the different variables could be directly compared (Hox, 2002; Hunter and Hamilton, 2002). As store sales were measured on level 2, this variable was standardized using grand mean centering.

#### Control Variables

The working population of the stores differed with regard to their educational level and age. Also the amount of hours the staff worked during the week was different across stores. In order to test the hypotheses, the control variables education, age, and hours worked were thus incorporated as covariates in the analyses.

### Analyses

A two-level mediation model was tested as employees were nested in stores and thus not statistically independent (Snijders and Bosker, 1994). Because store sales is assessed at level 2 and the mediators and dependent variable are assessed at level 1, a 2-1-1, cross-level, lower mediation model was required (Mathieu and Taylor, 2007). The intraclass correlation coefficients, depicting the amount of variance explained by grouping structure (Hox, 2002), were calculated using Raudenbush’s formula (1993). For work engagement, the ICC1 is 6.49%, for workload this value is 16.44%, and for job variety a value of 9.31% is found. These values imply that multilevel analysis was indeed appropriate (ICC1 > 0.05 as in Hox, 2002; Heck et al., 2013).

Three sets of hierarchical or mixed models were run in SPSS, with job variety, workload, and work engagement as dependent variables, respectively. The maximum likelihood function was left unrestricted (full information) in order to compare fixed effects in nested models (Hox, 2002; Heck et al., 2013). To verify whether more complex models provided better fit, chi-square (χ2) difference tests were performed. Additionally, proportional reductions in variance (PRV) components were inspected, as well as R-squared changes in individual and mean group scores (Snijders and Bosker, 1994).

For both mixed model analyses, a model with a random intercept effect was first examined (M1). Secondly, the control variables were added (M2). Lastly, the independent variables were added (M3). Finally, in line with the guidelines for mediation by MacKinnon et al. (2007), Sobel-tests (Sobel, 1982) were carried out.

## Results

### Descriptive Statistics and Correlations

Table 1 shows the means, SDs, and correlations at the individual level of analysis. One should note that the correlations of store sales are biased upward because they are not corrected for the smaller sample size at the second level of analysis. As expected, store sales was positively associated with workload (*r* = 0.191; *p* < 0.01) and negatively with job variety (*r* = −0.103; *p* < 0.05). Work engagement was significantly associated with job variety (*r* = 0.519; *p* < 0.01), whereas its negative association with workload was insignificant (*r* = −0.010; *p* > 0.05). The correlation between store sales and work engagement was insignificant (*r* = −0.072; *p* > 0.05).

**Table 1:** Means, SDs, and correlations on the individual level of analysis.

S. No.		μ	σ	1	2	3	4	5	6	7	8
1.	Store sales^1^ ^,^ ^2^	0.000	1.000								
2.	Job variety^1^	0.000	1.000	−0.103^*^	(0.786)						
3.	Workload^1^	0.000	1.000	0.191^**^	−0.010	(0.855)					
4.	Work engagement^1^	0.000	1.000	−0.072	0.519^**^	−0.048	(0.951)				
5.	Low education^3^	0.324	0.468	0.049	0.032	0.066	0.056				
6.	High education^3^	0.244	0.430	−0.030	−0.156^**^	−0.073	−0.270^**^	−0.391^**^			
7.	Age (20− years)^4^	0.311	0.463	0.036	−0.080	−0.231^**^	−0.134^**^	−0.037	−0.071		
8.	Age (30+ years)^4^	0.293	0.456	−0.069	0.131^**^	0.191^**^	0.229^**^	0.234^**^	0.305^**^	−0.432	
9.	Hours worked^5^	0.417	0.494	0.028	0.131^**^	0.197^**^	0.164^**^	0.067	0.297^**^	−0.342^**^	−0.142^**^

Cronbach’s Alpha of scales is reported between brackets. Level 1 sample size = 525. Level 2 sample size = 101.

1 Higher values equal higher scores on the construct.

2 Significance tests do not take into account reduced sample on the second level.

3 Dummy variables with medium education as referent group.

4 Dummy variables with age 20–29 years as referent group.

5 Dummy variables with less than 21 h per week as referent group.

* *p* < 0.05 (two-tailed);

** *p* < 0.01 (two-tailed).

### Comparison With Dutch and Sectoral Norms

In order to fully understand the specificity of our research context, we compared the descriptives of our sample to norms in the Netherlands and norms in the retail sector. It is found that employees showed an average engagement score of 5.435 (SD = 1.155) on a seven-point scale. Compared to Dutch norms, this average fell right below the upper limit of the “averagely engaged” category. Its exact position lay around the 74th percentile, meaning the sales employees in this organization were fairly engaged (Schaufeli and Bakker, 2003). An average job variety of 2.773 (SD = 0.590) was reported. This score laid around the 35th percentile compared to the general Dutch norm and around the 39th percentile of the norm in the Dutch food/non-food retail sector (SKB, 2014). Seemingly, the job variety experienced by this sample was slightly lower than one would expect. Finally, employees reported an average workload of 2.051 (SD = 0.564). This was lower than the Dutch norm and also lower than the experienced workload in the food/non-food retail sector of the Netherlands. Respectively, this current sample resided at either the 8th or the 5th percentile (SKB, 2014) indicating a notably low workload.

### Work Engagement on Store Sales, Job Variety, and Workload

To test Hypotheses 1 and 2, three nested models were ran as can be seen in Table 2. In M1, the intercept was inserted as a random factor so that it could vary between stores, explaining 0.35% of the variance in the engagement of individuals (−2LL = 1379.295). In M2, the control variables were added as predictors and the model explained significantly more variance than its predecessor (χ2 = 61.655; df = 5; *p* < 0.001). Finally, in M3, store sales, job variety and workload were added as predictors. This model predicted work engagement significantly better (χ2 = 143.028; df = 2; *p* < 0.001), explaining over a third of the variance in the individual scores and over 40% of variance in the mean group scores on work engagement. Only 2.11% of the total variance resided at the second level after this model step, indicating that job variety and workload accounted for almost all of the variance in mean group scores. Job variety was strongly positively related to engagement (*B* = 0.463; *p* < 0.001); workload had a smaller, negative effect (*B* = −0.098; *p* < 0.01). To conclude, a positive effect of job variety and a negative effect of workload on work engagement is found, supporting Hypotheses 1 and 2.

**Table 2:** Predicting work engagement.

	M1	M2	M3
−2LL	1482.838	1420.728^***^	1275.456^***^
χ^2^		61.655	143.028
df	2	5	2
Intercept	0.011 (0.050)	0.085 (0.112)	0.099 (0.096)
Low education^1^		−0.124 (0.097)	−0.101 (0.084)
High education^1^		−0.543 (0.118)^***^	−0.442 (0.103)^***^
Age (under 20)^2^		−0.173 (0.110)	−0.167 (0.097)
Age (30 and over)^2^		0.302 (0.109)^**^	0.231 (0.097)^*^
Hours worked^3^		0.127 (0.095)	0.061 (0.084)
Store sales			−0.001 (0.039)
Job variety			0.463 (0.037)^***^
Workload			−0.098 (0.038)^**^
Estimates of variance components			
First: individual (σ^2^)	0.923 (0.063)^***^	0.813 (0.056)^***^	0.651 (0.045)^***^
Second: store (τ^2^)	0.074 (0.036)^*^	0.075 (0.037)^*^	0.014 (0.023)
Intraclass coefficient (%)	7.42	8.45	2.11
Estimated modeled variance			
R^2^_1_ (%)	0.35	11.25	33.54
R^2^_2_ (%)	0.10	8.33	43.81

Effect values are unstandardized parameter estimates, with standard errors between brackets.

1 Dummy variables with medium education as referent group.

2 Dummy variables with age 20–29 years as referent group.

3 Dummy variables with less than 21 h per week as referent group.

* *p* < 0.05 (two-tailed);

** *p* < 0.01 (two-tailed);

*** *p* < 0.001 (two-tailed).

### Job Variety on Store Sales

Table 3 includes the three models with job variety as dependent variable. In M1, the intercept was inserted as a random factor so that it could vary between stores (−2LL = 1479.989). This did not explain notable variance in the individual scores of job variety nor in the group means, nor did the variance components differ significantly from the empty model. Subsequently, in M2, the individual differences were entered as control variables but these did not have a significant effect on the individual job variety and this model did not predict job variety significantly better than the previous M1 (χ2 = 21.338; df = 5; *p* < 0.001). In M3, store sales were added as a predictor. This was an improvement over M2 (χ2 = 4.369; df = 1; *p* < 0.05). Sales had a significant negative effect on job variety (*B* = −0.107; p < 0.05) and this provided evidence to confirm Hypothesis 3, which states that employees in retail stores with high sales experience less variety in their jobs.

**Table 3:** Predicting job variety.

	M1	M2	M3
−2LL	1479.989	1458.651^***^	1454.282^*^
χ^2^		21.338	4.369
df	2	5	1
Estimates of fixed effects			
Intercept	0.007 (0.051)	−0.034 (0.117)	−0.035 (0.116)
Low education^1^		−0.054 (0.100)	−0.046 (0.100)
High education^1^		−0.217 (0.122)	−0.228 (0.121)
Age (under 20)^2^		−0.087 (0.114)	−0.085 (0.114)
Age (30 and over)^2^		0.175 (0.113)	0.159 (0.113)
Hours worked^3^		0.191 (0.098)	0.195 (0.098)^*^
Store sales			−0.107 (0.051)^*^
Estimates of variance components			
First: individual (σ^2^)	0.904 (0.062)^***^	0.865 (0.060)^***^	0.864 (0.060)^***^
Second: store (τ^2^)	0.093 (0.040)^*^	0.094 (0.040)^*^	0.084 (0.039)^*^
Intraclass coefficient (%)	9.33	9.80	8.86
Estimated modeled variance			
R^2^_1_ (%)	−0.03	3.77	4.66
R^2^_2_ (%)	0.22	2.75	6.20

Effect values are unstandardized parameter estimates, with standard errors between brackets.

1 Dummy variables with medium education as referent group.

2 Dummy variables with age 20–29 years as referent group.

3 Dummy variable with less than 21 h per week as referent group.

* *p* < 0.05 (two-tailed);

*** *p* < 0.001 (two-tailed).

### Workload on Store Sales

With workload as a dependent variable, Hypothesis 4 was tested. M1 in Table 4 presents the addition of randomly varying intercepts between stores (−2LL = 1470.657). This model accounted for a minor 0.31% of individual variance and 1.60% of stores’ reported mean workload. M2, including covariates, modeled workload significantly better than M1 (χ2 = 58.918; df = 5; *p* < 0.001). M3 included store sales as a predictor of workload and fitted the data significantly better than M2 (χ2 = 12.679; df = 1; *p* < 0.001). This model supports Hypothesis 4 as it demonstrates that store sales has a positive effect on the workload of employees in that store (*B* = 0.197; *p* < 0.01).

**Table 4:** Predicting workload.

	M1	M2	M3
−2LL	1470.657	1411.739^***^	1399.060^***^
χ^2^		58.918	12.679
df	2	5	1
Estimates of fixed effects			
Intercept	−0.004 (0.055)	−0.127 (0.114)	−0.115 (0.112)
Low education^1^		0.027 (0.095)	0.022 (0.095)
High education^1^		−0.019 (0.116)	0.008 (0.115)
Age (under 20)^2^		−0.336 (0.108)^**^	−0.331 (0.108)^**^
Age (30 and over)^2^		0.274 (0.107)^*^	0.293 (0.106)^**^
Hours worked^3^		0.310 (0.093)^**^	0.294 (0.092)^**^
Store sales			0.197 (0.053)^**^
Estimates of variance components			
First: individual (σ^2^)	0.856 (0.059)^***^	0.737 (0.051)^***^	0.739 (0.051)^***^
Second: store (τ^2^)	0.142 (0.046)^**^	0.180 (0.048)^***^	0.138 (0.042)^**^
Intraclass coefficient	14.23	19.63	15.74
Estimated modeled variance			
R^2^_1_ (%)	0.31	8.40	12.39
R^2^_2_ (%)	1.60	−2.40	10.33

Effect values are unstandardized parameter estimates, with standard errors between brackets.

1 Dummy variables with medium education as referent group.

2 Dummy variables with age 20–29 years as referent group.

3 Dummy variable with less than 21 h per week as referent group.

* *p* < 0.05 (two-tailed);

** *p* < 0.01 (two-tailed);

*** *p* < 0.001 (two-tailed).

### Mediation via Job Characteristics

To test the mediation hypotheses, the requirements for mediation by MacKinnon et al. (2007). These prescribe that in order for mediation to occur (1) the independent variable needs to be significantly related to the mediator and (2) the mediator needs to be significantly related to the dependent variable. Both requirements were met as store sales was significantly related to job variety as well as workload (M3, Tables 3 and 4) and both job variety and workload had significant effects on work engagement (M3, Table 2). Hence, two Sobel-tests (Sobel, 1982) were conducted. These show that store sales had negative indirect effects on work engagement *via* job variety (B = −0.050; z = −2.069; *p* < 0.05) and *via* workload (*B* = −0.019; z = −2.12; *p* < 0.05). Although these effects are somewhat small, these indirect effects provide evidence for Hypotheses 5 and 6. Sales have no direct effect on work engagement as it traverses completely through job variety and workload.

## Conclusion and Discussion

This study focuses on examining the potential costs of increasing store sales for sales employees. Understanding the relationship between store sales and work engagement in a retail setting is important given the economic stagnation and recession that this context was faced with at that time. As stores are pressured to increase revenue and store sales, it is crucial to examine the implications of this on workers. Two negative cross-level indirect effects between store sales and work engagement were identified. More specifically, higher sales of retail stores resulted in lower levels of work engagement among retail sales employees due to the lower levels of job variety and elevated levels of workload it created. This finding is crucial as it shows that there are indeed costs associated with increases in store sales. Higher levels of store sales impact employees’ experience of work, resulting in lowered levels of work engagement. In line with work intensification theory, retail organizations aiming for better operational performance tend to make work more intense. Without concurrent investments, this might ultimately harm the engagement of their sales employees and be detrimental to long-term organizational viability.

Our results show that store sales levels have a significant negative effect on sales employees’ perceived job variety. High sales in this context results in a larger number of customers for employees to attend to. Likely, it results in a narrow focus on value-adding tasks, like working the register and restocking the store. In less productive stores, employees perceived more opportunities to spend their time on a larger diversity of tasks, like welcoming the customers, training their sales skills, familiarizing new employees, or improving the store layout.

Our finding that store sales were associated with higher levels of workload can be understood in the context of stagnation and recession of the Dutch retail market (CBS, 2014). Stores that achieved high revenues might not have the means to increase headcount as a way to alleviate these pressures. Staff increases would have reduced the pressures on employees but would also decrease the competitive advantage needed in the economic climate at that time. Thus, in order to maintain this high sales, store managers have demanded increasingly high levels of focused effort from their employees. Looking at the recent economic climate, we see that the retail sector has shown a small growth in financial turnover figures with an average growth rate of 1.4% per year (CBS, 2020). Although this rate of growth is slightly higher than it was in the years before 2013, we do not expect this to change our results. If anything, one could expect that this slightly larger growth has increased the work intensification and consequent negative indirect effect of store sales on engagement. This finding thus reinforces the conclusions of our study and demonstrates the importance of our results today.

We have shown that there are complex processes at play between store sales and productivity. In the current study, two simultaneous negative pathways were identified which provide insight into the processes through which store sales affect levels of work engagement, thereby shedding light on how job characteristics shape the relationship between store sales and employee engagement. These findings provide evidence for the applicability of work intensification theory in a retail setting, as high performance seems to create a work environment unfavorable to engagement levels.

The findings of this study challenge theories linking job demands, resources, engagement, and performance. Based on the theory by Hobfoll (2011), one could argue that these elements will strengthen each other, as resources will create more resources which will, in turn, lead to higher engagement and performance, resulting again in more resources. The current study however challenges this type of positive gain spiral in two ways. We demonstrated that in the retail context, store sales is related to (1) less varied jobs and (2) higher levels of job demands. These findings present a more complex relationship between job demands, resources, work engagement, and sales, and suggest that this relationship depends on the nature of the context. Future studies could examine these processes in more detail and the role of various types of demands and resources should be examined in more depth. For example, it could be studied to what extent sales affects autonomy (job resource) or emotional demands (job demand), which may further explain the relationship between store sales and work engagement.

Our study further shows that it is crucial to look at the implications higher sales has for employees in terms of demands and resources, if one wants to understand how store sales relates to employee outcomes. In the current research context, high store sales means that employees need to use a larger proportion of their time to carry out a limited number of tasks, thus decreasing their job variety. Also, longer queues at the register result in higher workload. In this specific setting increased sales immediately impacted the type and amount of tasks that needed to be performed by employees. In other settings, increased sales might not be associated with higher workload. On the contrary, in some settings, higher sales might result in additional resources being provided to employees. With this study, we help extend research on store sales and engagement by showing that this relationship is dependent on the specific organizational context that either reacts with more job demands (in our case) or with more job resources.

### Limitations and Future Research Implications

While the current study makes use of both survey and objective financial data, the cross-sectional nature of the data prevents definite conclusions regarding causality between the constructs. Although theory as well as the specific context that we have studied supports the hypothesized direction of the relationships, future studies are advised to examine these relationships based on a longitudinal research design.

Secondly, this study is performed within stores that are part of a retail chain in the Netherlands. This might limit the generalizability of the results. For instance, employees report relatively low levels of workload compared to other Dutch retail employees. Future studies should examine whether the findings can be generalized to this sector. Moreover, even though response rates were substantial (27.03%), participation was voluntary and selection effects should therefore be considered. The objective data does not suggest the existence of selection effects, however, whether non-participation was related to the perceptions and attitudes of employees cannot be determined. Finally, the retail chain, we studied employed mainly women. Future studies would do well to explore retailers with a more diverse workforce and study the extent to which our current results hold in those other contexts.

Third, while this study included job variety and workload, other variables might further explain employees’ experience of work engagement. It is therefore recommended that future studies examine a larger set of job resources and demands. Employee perceptions of feedback and autonomy would be welcome additions as task-related resources are considered important determinants of employee attitudes (Halbesleben, 2010). Future research could examine if, and which, job demands form job challenges in a retail setting, as the current study found that workload may hinder work engagement instead of encouraging it. Relatedly, it could prove useful to examine job demands and resources on the unit level as such store-level variables can explain differences above and beyond individual-level predictors (Piening et al., 2013). Moreover, it would be insightful for future research to examine whether the effects of lower job variety on work engagement differ when the resulting set of tasks is perceived as more or less gratifying by the employee. Additionally, it could be studied to what extent the effects might be related to management style, and whether and how managers could mitigate the negative effects of job variety and workload on work engagement. Finally, it would be insightful to study whether store sales affect the extent to which store managers are able to provide feedback to employees or discuss developmental opportunities with employees (both job resources). Higher sales could be at the expense of implementing such activities by store managers, and lower engagement levels could result.

### Practical Implications

The findings of this study suggest that in this specific retail organization management should pay close attention to maintaining high levels of store sales, while at the same time making sure that personnel can cope with increased workload and unchallenging work. Especially in rough times, in which cost containment and workforce reduction are part of the leading strategies in retail, it is important that retail organizations remain legitimately viable as well. We therefore plea for a more balanced approach in which the desire for financial success is combined with a concern for healthy employees (Paauwe and Farndale, 2017). Redesigning work so that more job variety is experienced will positively affect work engagement and as such contribute to employee health. Redesigning the work environment might also mitigate the negative effects we reported. More specifically, Neirotti (2020) argues that a supportive team environment can help mitigate the negative effects of work intensification (i.e., higher workload) on employee outcomes. Similarly, Mauno et al. (2019) argue that investing in more social support can protect employees against job strain and might even alter perceptions about how demanding the current job is, and as such function as a job resource (Giauque et al., 2019). When such a balanced approach is adopted, it is ensured that retail organizations do not only invest in creating financial gain but also in maintaining a resourceful and nourishing work environment for its employees.

## Data Availability Statement

The datasets for this article are not publicly available because the organization studied does not allow the data to be publicly available. Requests to access the datasets should be directed to SB, s.e.beijer@vu.nl.

## Ethics Statement

Ethical review and approval was not required for the study on human participants in accordance with the local legislation and institutional requirements. Written informed consent for participation was not required for this study in accordance with the national legislation and the institutional requirements.

## Author Contributions

PL: theorizing, data collection, data analysis, writing, and coordinating. SB: theorizing, data collection, writing, and coordinating. SN: theorizing and writing. MV: theorizing, data collection, data analysis, and writing. JP: theorizing. All authors contributed to the article and approved the submitted version.

### Conflict of Interest

The authors declare that the research was conducted in the absence of any commercial or financial relationships that could be construed as a potential conflict of interest.
